# Functional characterization of a putative DNA methyltransferase, EadM, in *Xanthomonas axonopodis* pv. *glycines* by proteomic and phenotypic analyses

**DOI:** 10.1038/s41598-019-38650-3

**Published:** 2019-02-21

**Authors:** Hye-Jee Park, Boknam Jung, Jungkwan Lee, Sang-Wook Han

**Affiliations:** 1 Department of Integrative Plant Science, Chung-Ang University, Anseong, 17546 Republic of Korea; 2 Department of Applied Biology, Dong-A University, Busan, 49315 Republic of Korea

## Abstract

*Xanthomonas axonopodis* pv. *glycines* (*Xag*) is a phytopathogenic bacterium causing bacterial pustule disease in soybean. Functions of DNA methyltransferases have been characterized in animal pathogenic bacteria, but are poorly understood in plant pathogens. Here, we report that functions of a putative DNA methyltransferase, EadM, in *Xag*. An EadM-overexpressing strain, *Xag*(EadM), was less virulent than the wild-type carrying an empty vector, *Xag*(EV). Interestingly, the viable cell numbers of *Xag*(EadM) were much lower (10-fold) than those of *Xag*(EV) at the same optical density. Comparative proteomic analysis revealed that proteins involved in cell wall/membrane/envelope and iron-transport were more abundant. Based on proteomic analysis we carried out diverse phenotypic assays. Scanning electron microscopy revealed abnormal bacterial envelopes in *Xag*(EadM). Additionally, *Xag*(EadM) showed decreased stress tolerance against ciprofloxacin and sorbitol, but enhanced resistance to desiccation. Exopolysaccharide production in *Xag*(EadM) was also decreased. Production of siderophores, which are iron-chelators, was much higher in *Xag*(EadM). As in *Xag*, *Escherichia coli* expressing EadM showed significantly reduced (1000-fold) viable cell numbers at the same optical density. Thus, EadM is associated with virulence, envelope biogenesis, stress tolerance, exopolysaccharide production, and siderophore production. Our results provide valuable and fundamental information regarding DNA methyltransferase functions and their related cellular mechanisms in plant pathogenic bacteria.

## Introduction

*Xanthomonas axonopodis* pv. *glycines* (*Xag*) is a Gram-negative bacterium causing bacterial pustule disease on soybean, which is one of the most serious diseases and that reduces the yield and quality of the crop^1^. This disease is widely distributed in most soybean-growing fields and, under favorable conditions, yield loss of the crop can reach 53%^2^. In Korea, the disease had been nationally found in up to 89.7% of soybean-cultivated areas^3^. *Xag* can penetrate soybean leaves through natural openings including stomata and wounds, and colonize in intercellular spaces^4^. Typical symptoms are small, light-colored pustules surrounded by chlorotic halos on the underside of soybean leaves^5^. The spots vary from specks to large and irregular brown areas.

Virulence mechanisms of *Xag* have been studied in previous decades and full genome sequences of *Xag* have been determined^6–8^. Previous studies focused on the type III secretion system and quorum sensing system to elucidate the virulence mechanisms. For example, HpaG, one of the type III effectors, is responsible for triggering a hypersensitive response in nonhost plants^9^. *Xag* mutants that cannot synthesize diffusible signal factors showed reduced virulence on soybean leaves^10^. In addition, the LuxR-type transcriptional regulator *Xag*R is associated with virulence^11^. However, the roles of DNA methyltransferases involved in virulence or other mechanisms have not been reported in *Xag*.

DNA methyltransferase is an enzyme which transfers methyl groups from *S*-adenosyl-L-methionine to specific nucleotides. In eukaryotic organisms, DNA methylation is well-understood and is known to have important roles in chromatin remodeling, genomic imprinting, gene expression, and embryonic development^12,13^. Furthermore, in *Arabidopsis thaliana*, DNA methylation and demethylation are involved in antagonistically regulating basal resistance against both biotrophic and necrotrophic pathogens^14^. Hypo-methylated mutants show enhanced disease resistance, but hyper-methylated mutants exhibit high susceptibility. In an opportunistic pathogen, *Aspergillus flavus*, a mutant lacking *dmtA* displayed abnormal phenotypes and declined formation of conidia^15^.

In bacteria, DNA methyltransferases have been well-studied as part of the restriction-modification system for protection against foreign DNA^16^. Additionally, bacterial DNA modified by methyltransferases are involved in virulence and diverse cellular mechanisms in animal-associated bacteria. In *Streptococcus mutans* which causes tooth decay, DNA methylation regulates the expression of mutacin production and virulence genes^17^. In addition, methylation by a DNA adenine methyltransferase is necessary for biofilm formation in *Salmonella enterica* serovar Enteritidis^18^. However, the functions of DNA methyltransferases are poorly understood in plant pathogenic bacteria.

To predict the functions of genes/proteins, comparative omics-based approaches including transcriptomics and proteomics have been employed. However, the expression of genes at the RNA level is not always correlated with the abundance of proteins because of posttranslational processes and regulation. For example, the correlation between RNA expression and protein abundance was only up to 50% in 23 human cell lines^19^. Among eight proteins associated with HrpX, which is a transcriptional regulator and indispensable for pathogenicity, identified by comparative proteomics, the RNA expression of only one gene was correlated with protein abundance in *Xanthomonas* spp.^20^. Therefore, proteomic analysis is increasingly used to understand cellular and biological mechanisms and proteomic approaches have been widely recognized as pivotal tools.

Here, we report functions of a putative DNA methyltransferase, EadM (putative envelope-associated DNA methyltransferase; Accession No., AOY64023), in *Xag* whose methylome has been determined^8^. We generated the *Xag* strain 8ra overexpressing EadM, *Xag*(EadM) and compared the protein abundance of *Xag*(EadM) with that of the wild-type carrying an empty vector, *Xag*(EV), using label-free shotgun proteomic analysis combined with clusters of orthologous groups (COGs). Based on the COG classification, we conducted diverse phenotypic assays. Proteomic characterization and phenotypic observation indicated that EadM is involved in virulence, envelope formation, stress tolerance, exopolysaccharide (EPS) production, and siderophore production. Finally, we demonstrated that the expression of EadM in *Escherichia coli* exhibited similar effects on growth as expression in *Xag*.

## Results

### EadM is involved in virulence and affects viable cell numbers of *Xag*

EadM possesses an *S*-adenosyl methionine-dependent methyltransferase domain and is highly homologous with putative DNA methyltransferases in closely related genera (Supplementary Fig. 1). To investigate the roles of EadM in virulence, we attempted to generate both the *eadM*-knockout mutant and EadM-overexpressing strain. However, the knockout mutant could not be generated, despite many attempts. Therefore, we used only the overexpressing strain, *Xag*(EadM), for all proteomic and phenotypic analyses. Before phenotypic assays, we checked the expression of *eadM* gene in *Xag*(EV) and *Xag*(EadM) using quantitative PCR (qPCR) (Supplementary Fig. 2). Transcripts of *eadM* gene in *Xag*(EadM) were significantly higher than that in *Xag*(EV), demonstrating that *Xag*(EadM) is indeed the EadM-overexpressing strain. *Xag* strains were infiltrated by needleless syringes on fully expanded trifoliate leaves of soybean at an optical density at 600 nm (OD_600 nm_) of 0.3. It is generally known that an OD_600 nm_ of 0.1 corresponds to 10^8^ cells/mL^21^. However, the levels of disease symptoms developed by *Xag* strains were very similar and impossible to quantify by naked eyes. Therefore, we quantified bacterial multiplication in the inoculated leaves. Firstly, we checked that the bacterial growth of *Xag* and *Xag*(EV) in soybean (Supplementary Fig. 3A). The average values of population from *Xag*(EV) is slightly lower than these from *Xag*, suggesting that there might be side effects from the vector in the later days. Therefore, we used *Xag*(EV), but not *Xag*, in all experiments.

**Figure 1:**
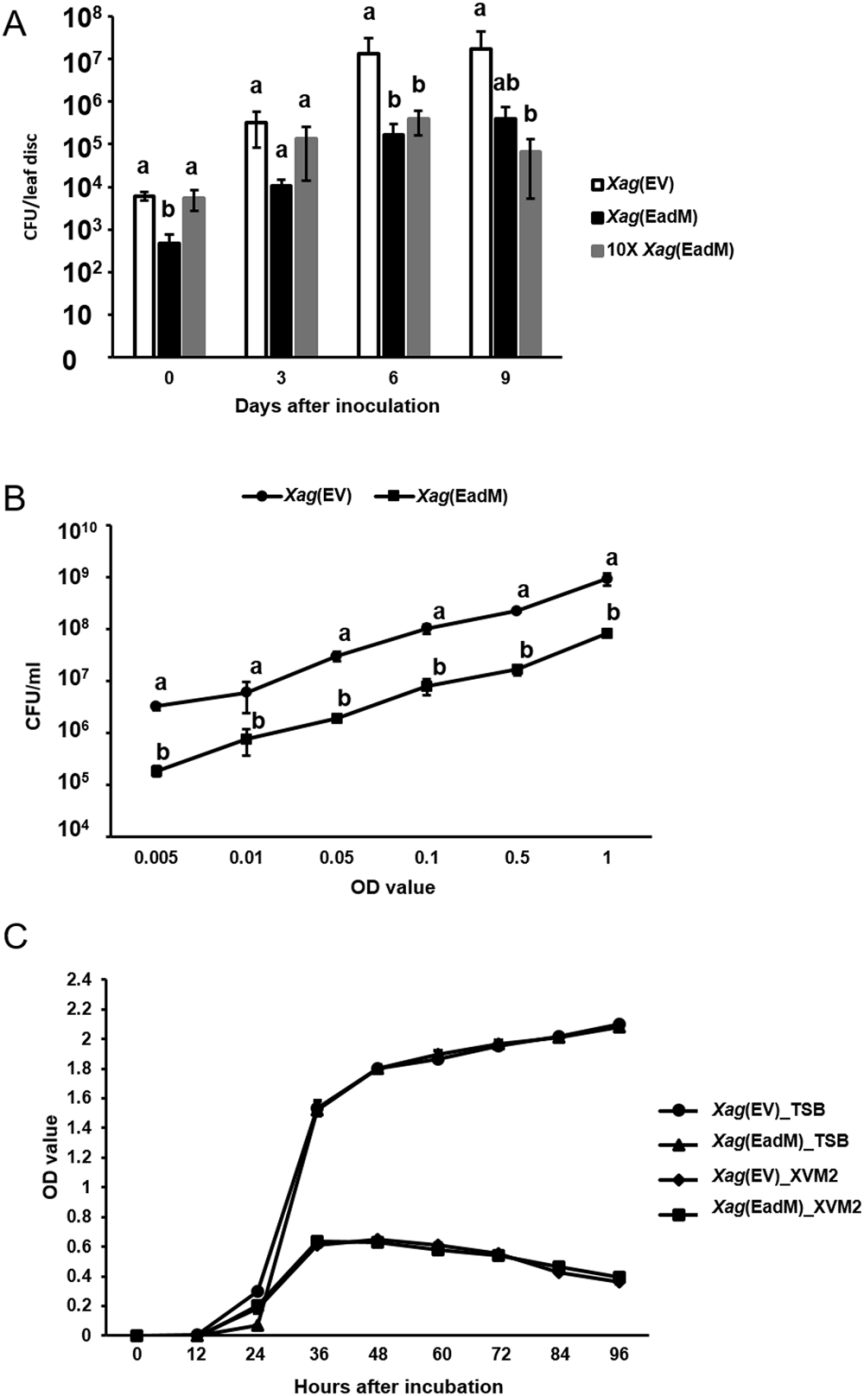
Measurement of bacterial population in plant and media for *Xag*(EV) and *Xag*(EadM). (**A**) Bacterial population of *Xag*(EV) (white), *Xag*(EadM) (black), and 10 × *Xag*(EadM) (grey) were measured by the colony counting method at 0, 3, 6, and 9 days after inoculation. (**B**) The viable cell numbers of *Xag*(EV) (circle) and *Xag*(EadM) (square) were quantified by the colony counting method at various optical density values using a spectrophotometer. (**C**) Bacterial growth of *Xag*(EV) in TSB (circle), *Xag*(EadM) in TSB (triangle), *Xag*(EV) in XVM2 (diamond) and *Xag*(EadM) in XVM2 (square) strains were established for 4 days at 12-h intervals. Different letters represent significant differences using the least significant difference test, P ≤ 0.05. Error bars represent the mean of three biological replicates with the standard deviations. All experiments were repeated at least three times with three biological replicates.

As shown in Fig. 1A, the population of *Xag*(EadM) was significantly lower than that of *Xag*(EV) at 3, 6, and 9 days after inoculation (DAI). Interestingly, the initial concentration of *Xag*(EadM) at 0 DAI was always lower (10-fold) than that of *Xag*(EV) in repeated experiments, although we used the same OD value for both strains. Therefore, we tested the virulence of *Xag*(EadM) using a 10-fold concentrated inoculum of the strain, 10 × *Xag*(EadM). At 0 DAI, the viable cell numbers counted as colony forming unit (CFU) from 10 × *Xag*(EadM) were similar to those of *Xag*(EV) (Fig. 1A). The 10 × *Xag*(EadM) displayed reduced viable cell numbers compared to *Xag*(EV) at 6 and 9 DAI, suggesting that EadM is involved in virulence in *Xag*.

Because the viable cell numbers of *Xag*(EV) and *Xag*(EadM) differed at 0 DAI, we compared the viable cell numbers of *Xag*(EadM) with *Xag*(EV) by measuring CFU at various OD values (Fig. 1B). The viable cell numbers of *Xag*(EadM) were significantly lower (10-fold) than those of *Xag*(EV) at all tested OD values (0.005–1), suggesting that overexpression of EadM in *Xag* interfered with the OD values. Additionally, we also tested viable cell numbers of *Xag* and *Xag*(EV) at various OD values (Supplementary Fig. 3B). There was no difference between *Xag* and *Xag*(EV). Next, we tested whether EadM is involved in bacterial growth using rich media, tryptic soy broth (TSB), and plant-mimic media, XVM2 (Fig. 1C). *Xag*(EV) and *Xag*(EadM) displayed nearly identical growth patterns in both media. It suggests that multiplication of *Xag* was not affected by EadM.

### Comparative proteomic analysis for postulating EadM function

It is clear that overexpression of EadM has negative effects on virulence and affects OD values. To predict the cellular and biological mechanisms associated with EadM, we carried out comparative proteomic analysis using *Xag*(EV) and *Xag*(EadM). Protein abundance in *Xag*(EV) was compared to that in *Xag*(EadM) using a label-free comparative shotgun proteomic approach followed by COG analysis to classify selected proteins.

**Figure 2:**
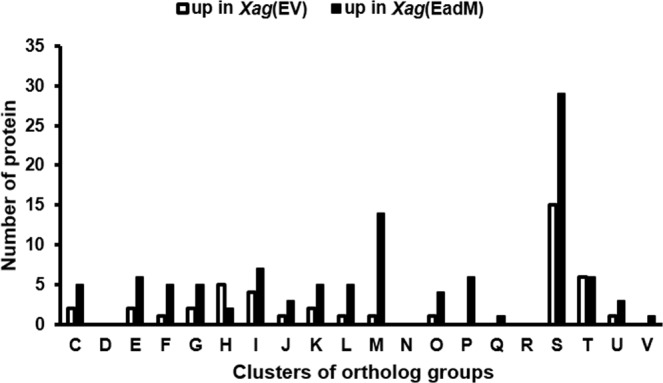
Clusters of orthologous group (COG) analysis of differentially abundant proteins in *Xag*(EV) and *Xag*(EadM). Bars represent COG groups of 43 and 106 proteins, which were differentially abundant (>2 fold) in *Xag*(EV) and *Xag*(EadM), respectively. Abbreviations: C, Energy production and conversion; D, Cell cycle control and mitosis; E, Amino acid metabolism and transport; F, Nucleotide metabolism and transport; G, Carbohydrate metabolism and transport; H, Coenzyme metabolism; I, Lipid metabolism; J, Translation; K, Transcription; L, Replication and repair; M, Cell wall/membrane/envelop biogenesis; N, Cell motility; O, Post-translational modification, protein turnover, chaperone functions; P, Inorganic ion transport and metabolism; Q, Secondary structure; R, General functional prediction only; S, Function unknown; T, Signal transduction; U, Intracellular trafficking and secretion; V, Defense mechanisms.

The numbers of detected proteins and peptide spectral matches from three biological replicates of *Xag*(EV) and *Xag*(EadM) are shown in Supplementary Table 1. Total of 1013 and 1078 proteins were common in the three biological replicates of *Xag*(EV) and *Xag*(EadM), respectively (Supplementary Table 1). At least 92.8% of the detected proteins from one biological replicate belonged to the shared proteins that had been commonly found in the three biological replicates, indicating that sample preparation and liquid chromatography-tandem mass spectrometry analysis were effectively carried out. The proteins were used for comparative analysis. Among them, 43 and 106 proteins were more abundant (over 2-fold) in *Xag*(EV) and *Xag*(EadM), respectively (Supplementary Tables 2 and 3), and these differentially abundant proteins were categorized by COG analysis (Fig. 2). The number of categorized proteins of *Xag*(EadM) was higher than that of *Xag*(EV) in most categories of COGs except for group H (coenzyme transport and metabolism) (Fig. 2). Interestingly, proteins belonging to group M (cell wall/membrane/envelope biogenesis) were the most abundant and were outer membrane-related proteins including outer membrane protein assembly factor, lipid A biosynthesis lauroyl acyltransferase, OmpW, GumB, LolA, lipid-A-disaccharide synthase, LptA, and YidC. In addition, many iron-related proteins including AcnD, NfuA, ferric enterobactin receptor, 3 of TonB-dependent receptors, SufE, and HseB, were detected.

### EadM is involved in bacterial envelope development of *Xag*

**Figure 3:**
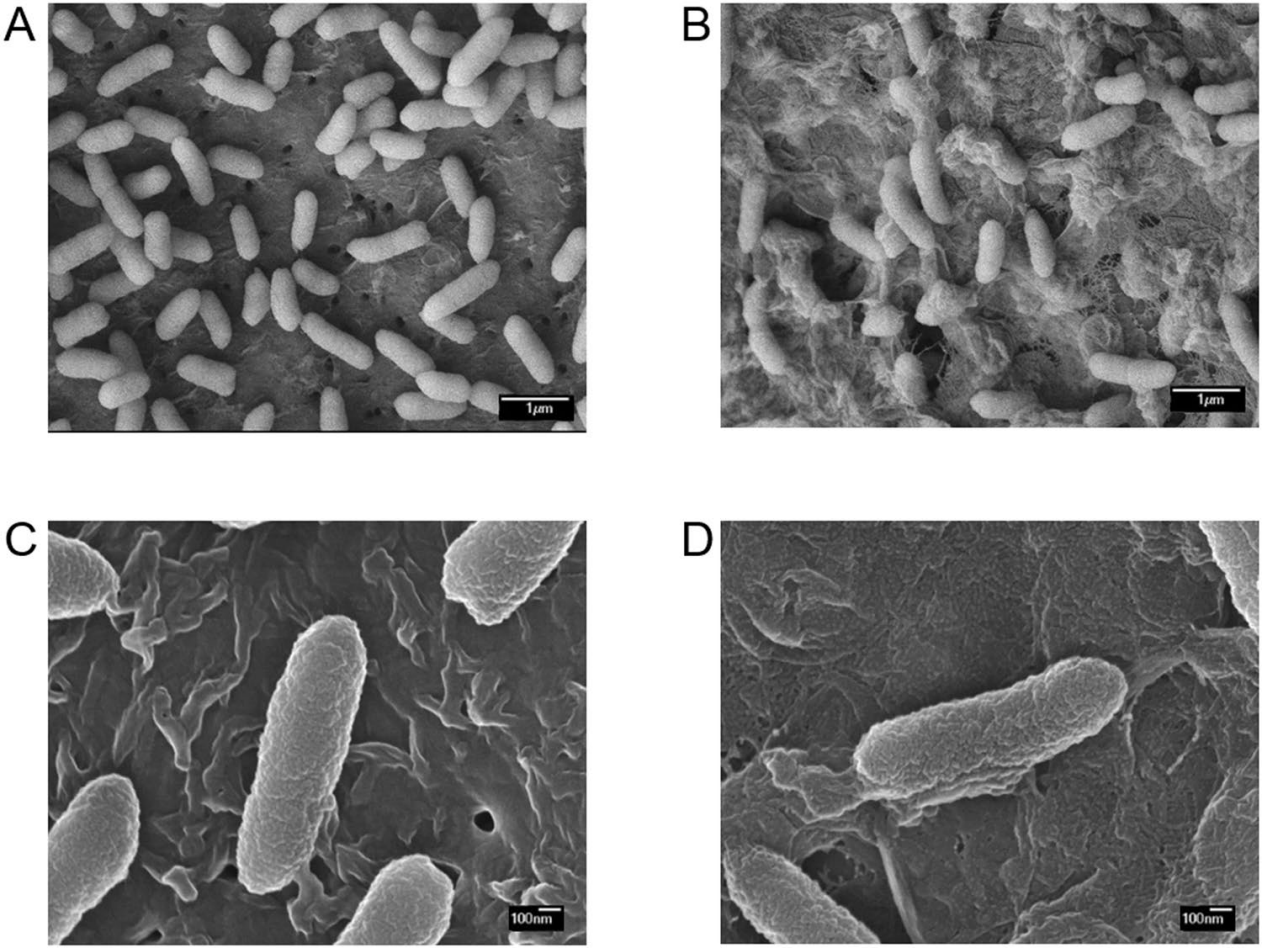
Scanning electron micrographs of *Xag* strains. Morphologies of *Xag*(EV) (**A**,**C**) and *Xag*(EadM) (**B**,**D**) strains incubated for 24 h in TSB at 28 °C were observed with a scanning electron microscope with a JSM-6700F microscope (Jeol). Size bars represent 1 μm and 100 nm.

Because bacterial wall/membrane/envelope-associate proteins were the most abundantly identified proteins in the proteomic analysis and EadM interfered with OD values, we examined the morphology of *Xag*(EV) and *Xag*(EadM) grown in TSB medium using a scanning electron microscope (Fig. 3). The size and rod-like shape of both strains did not differ, but *Xag*(EadM) showed abnormal materials, which might be bacterial envelopes or materials from cell lysis, compared to *Xag*(EV). The envelopes of *Xag*(EV) were intact and the abnormal materials from the bacterial cells were rarely found (Fig. 3A). However, the putative envelopes of *Xag*(EadM) were peeled from the bacterial cells and the putative peeled envelopes were nested and overlapped on the mounting materials (Fig. 3B). In addition, stretched materials from *Xag*(EadM) were clearly observed, but not from *Xag*(EV) (Fig. 3C,D). This observation reveals that EadM is associated with the envelope tightness/development and that putative peeled envelopes from *Xag*(EadM) interfere with OD values, reducing viable cell numbers.

### Overexpression of EadM negatively affects tolerance to ciprofloxacin and D-sorbitol as well as EPS production but has positive effects on desiccation

**Figure 4:**
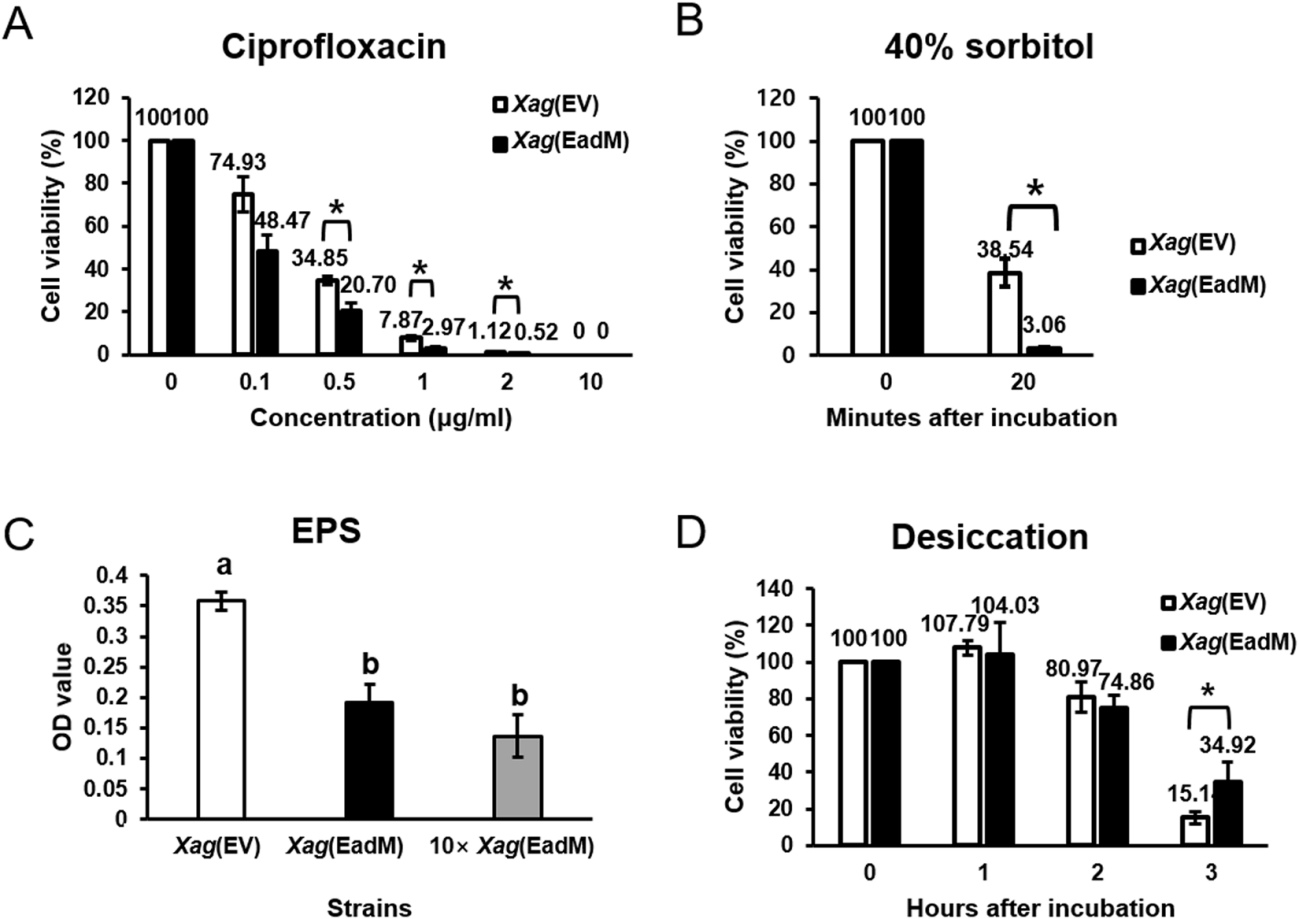
Tolerance to ciprofloxacin, sorbitol, desiccation, and EPS production of *Xag* strains. *Xag*(EV) (white) and *Xag*(EadM) (black) were exposed to (**A**) 0, 0.1, 0.5, 1, 2, and 10 μg/mL of ciprofloxacin for 2 h or (**B**) 40% sorbitol for 20 min. Bacterial cells were enumerated by a colony counting method. Viability was calculated by comparing the viable cell numbers in before and after treatment. (**C**) EPS production of *Xag* strains was evaluated using a phenol-sulfuric acid method. (**D**) *Xag*(EV) (white) and *Xag*(EadM) (black) were exposed to desiccation stress for 1, 2, and 3 h. Error bars represent the mean of three biological replicates with the standard deviations. Star marks on the bars represent significant differences (using the Student’s *t*-test, P ≤ 0.05). All experiments were repeated at least three times with three biological replicates.

Bacterial capsules have been recognized for protecting bacteria against the external environment. Our comparative proteomic analysis revealed that EadM is related to bacterial wall/membrane/envelope biogenesis functions and *Xag*(EadM) showed abnormal bacterial envelopes. Therefore, we presumed that the tolerance of *Xag*(EadM) against to external stresses would be altered. To test this hypothesis, we performed stress tolerance assays (Fig. 4). When ciprofloxacin (0, 0.1, 0.5, 1, 2, and 10 μg/mL), an antibiotic that eradicates microbes, was used to cultures of *Xag* strains for 2 h, the viability of *Xag*(EadM) was significantly lower than these of *Xag*(EV) in the given conditions compared to untreated controls (Fig. 4A). In the presence of 10 μg/mL of ciprofloxacin, both strains were not survived. Using the obtained values, the half maximal inhibitory concentration (IC50) was calculated. The values of IC50 in *Xag*(EV) and *Xag*(EadM) was 0.106 and 0.276 μg/mL, respectively. This indicates that *Xag*(EadM) is more sensitive (approximately 2.7-fold) than *Xag*(EV) (Fig. 4A). The peptidoglycan layer, a major component of the bacterial cell envelope, protects the bacterial cell against osmotic pressure^22^. Therefore, we predicted that *Xag*(EadM) with unstable cell envelopes would show altered viability under osmotic stress conditions. Following exposure to 40% D-sorbitol, an osmotic stress agent, for 20 min, *Xag*(EadM) showed significantly reduced viability (over 12-fold) compared to *Xag*(EV) (Fig. 4B).

In addition to envelopes, bacterial exopolysaccharides (EPS) possess protective functions against diverse environmental conditions including chemical agents^23^. Therefore, we performed an EPS production assay to determine whether EadM is involved in EPS formation (Fig. 4C). EPS production was assessed by measuring carbohydrates from EPS as described previously^24^. Because the method depends on the measurement of OD values after partial purification, we tested *Xag*(EadM) as well as 10 × *Xag*(EadM). As shown in Fig. 4C, *Xag*(EV) displayed higher absorbance compared to *Xag*(EadM) and 10 × *Xag*(EadM). The average OD value in *Xag*(EV) was 0.35, but this value in *Xag*(EadM) or 10 × *Xag*(EadM) was 0.2 or 0.15, respectively. Thus, overexpression of EadM in *Xag* negatively affected EPS production, indicating that EadM is involved in EPS formation. It is also known that EPS protects microorganisms from desiccation stress^25^. Therefore, we investigated the tolerance of *Xag*(EV) and *Xag*(EadM) to desiccation by measuring CFUs under desiccation conditions (Fig. 4D). Exposure to air for 1 and 2 h was not enough to completely dry bacterial cells, and the viability of *Xag*(EV) and *Xag*(EadM) was not statistically different in the conditions. In 3 h after incubation, the viability of *Xag*(EV) and *Xag*(EadM) was 15.1 and 34.92%, respectively, indicating that *Xag*(EadM) is more resistant to desiccation stress compared to *Xag*(EV) although the strain displayed reduced EPS production.

### EadM is involved in siderophore production

**Figure 5:**
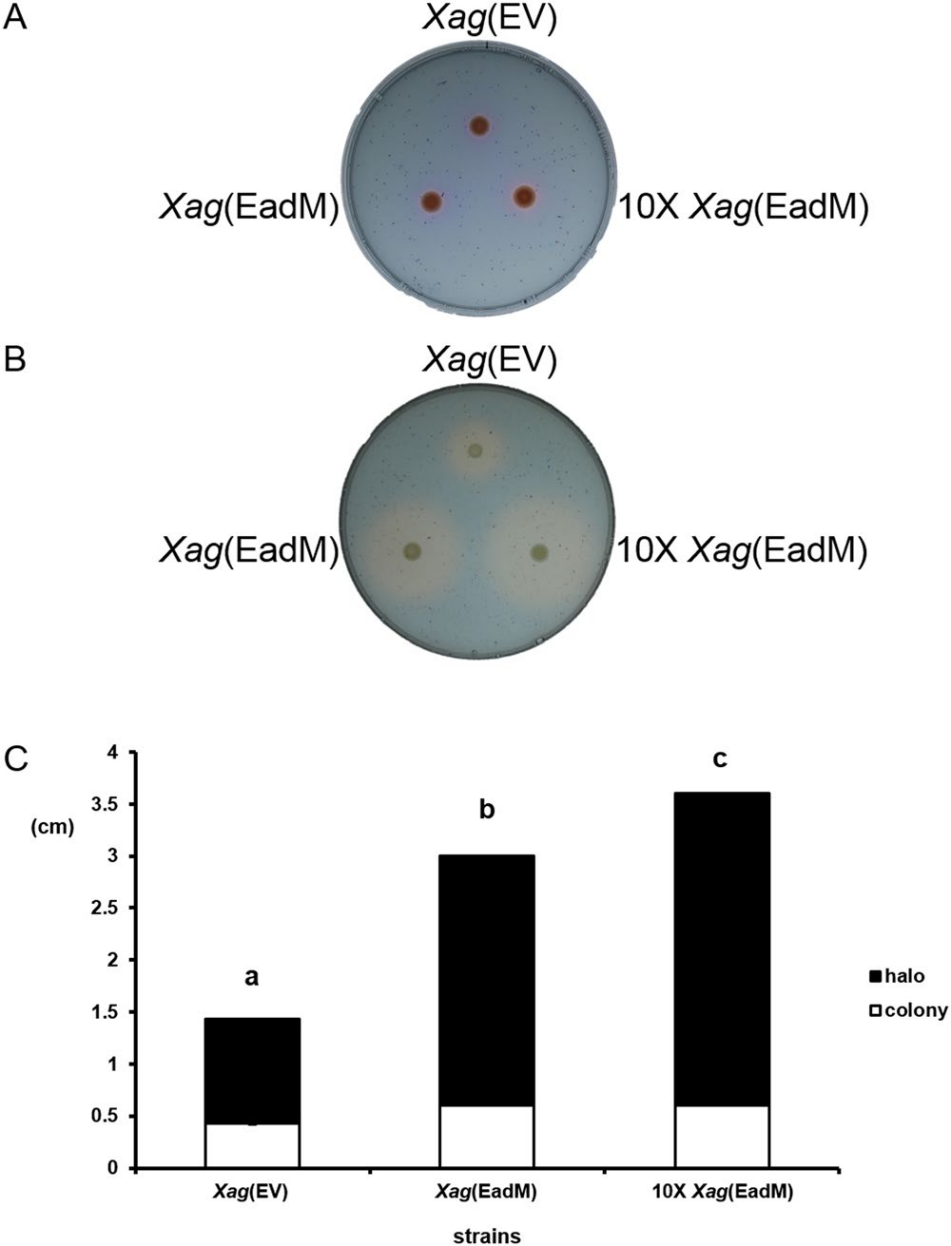
Measurement of siderophore production using chrome azurol sulfonate (CAS) assay for *Xag* strains. The halo from *Xag* strains were observed (**A**) under iron-rich conditions on a XVM2-CAS plate or (**B**) under iron-deficient conditions on a XVM2-CAS-BP plate. (**C**) Sizes of colonies (white) and siderophore halo zones (black) among *Xag* strains were measured at 3 days after incubation. Error bars represent the mean of three biological replicates with standard deviations. Different letters represent statistical difference using the least significant difference test, P ≤ 0.05. All experiments were repeated at least three times with three biological replicates.

Bacteria produce the iron-chelating compound siderophore to take up iron from extracellular environments^26^. Because diverse iron-related proteins were found in the proteomic analysis, EadM was predicted to be involved in iron-related mechanisms. Thus, we carried out a chrome azurol S (CAS) assay to assess siderophore production. In this assay, we used *Xag*(EV), *Xag*(EadM) and 10 × *Xag*(EadM) because the assay is dependent on measuring the diameter of halos produced by siderophores, but not CFU. Under iron-rich conditions on the XVM2-CAS-agar plate, there were no differences in colony and halo sizes among *Xag*(EV), *Xag*(EadM), and 10 × *Xag*(EadM) (Fig. 5A). However, halos from *Xag*(EadM) and 10 × *Xag*(EadM) were dramatically increased compared to that of *Xag*(EV) under iron-deficient conditions on the XVM2-CAS-BP-agar plate (Fig. 5B). The halo size of *Xag*(EV) was 1 cm, while those of *Xag*(EadM) and 10 × *Xag*(EadM) were 2 and 2.5 cm, respectively (Fig. 3C). However, the sizes of the colonies were nearly identical. These data indicate that overexpression of EadM enhances siderophore production under iron-deficient conditions.

### Expression of EadM reduces viable cells in *E. coli*

**Figure 6:**
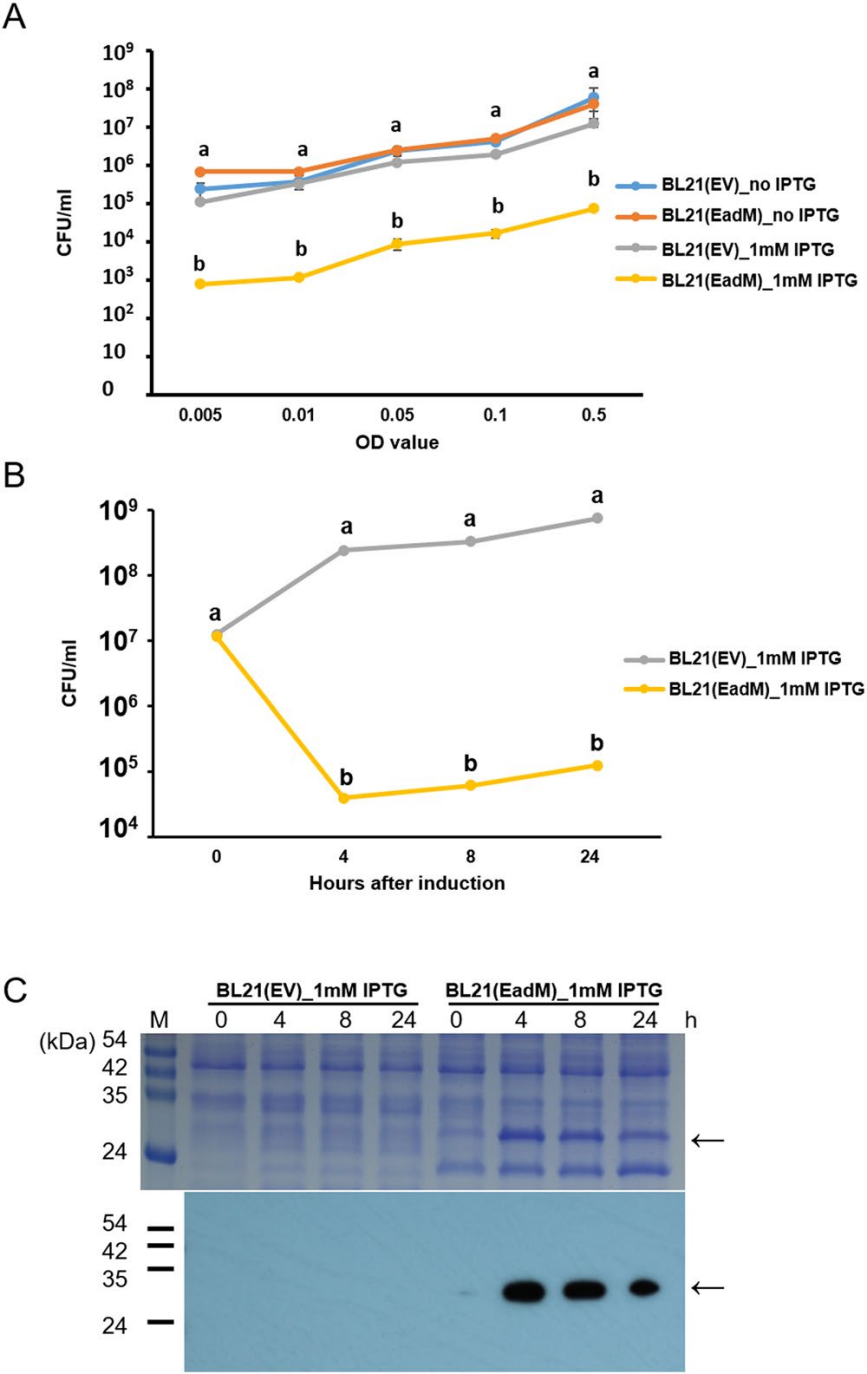
Effect of EadM expression in *E. coli* BL21. (**A**) Viable cell numbers of BL21(EV)_no IPTG (blue), BL21(EadM)_no IPTG (orange), BL21(EV)_IPTG (grey), and BL21(EadM)_IPTG (yellow) strains were measured by a colony counting method at various OD values. (**B**) After IPTG induction, the viable cell numbers of BL21(EV) (grey) and BL21(EadM) (yellow) were calculated at 0, 4, 8, and 24 h after incubation. Error bars represent the mean of three biological replicates with the standard deviations. Different letters represent statistical difference using the least significant difference test, P ≤ 0.05. All experiments were repeated at least three times with three biological replicates. (**C**) Expression of EadM protein in *E. coli* BL21 was confirmed by immunoblotting using an anti-6xHis antibody. Arrows indicate EadM protein.

We attempted to purify the EadM protein from the *E. coli* strain BL21 and pOPINF vector. However, we failed to obtain purified EadM. Because overexpression of EadM in *Xag* triggered reduced viable cell numbers at the same OD values (Fig. 1B), we examined the viable cell numbers of *E. coli* BL21 carrying pOPINF-EadM, BL21(EadM), with or without 1 mM isopropyl β-D-1-thiogalactopyranoside (IPTG); *E. coli* BL21 containing pOPINF, BL21(EV), was used as a negative control (Fig. 6A). There was no significant difference in viable cell numbers in BL21(EadM) without IPTG and BL21(EV) with or without IPTG at various OD values. However, viable cell numbers of BL21(EadM) in the presence of 1 mM IPTG were significantly reduced (1000-fold) compared to under other conditions, demonstrating that overexpression of EadM in BL21 has similar effects compared in *Xag* although *E. coli* and *Xag* are not closely related. Next, we evaluated the viable cell numbers of BL21(EadM) and BL21(EV) after treatment with 1 mM IPTG (Fig. 6B). Two strains displayed similar viable cell numbers at 0 h after induction. As expected, the viable cell numbers of BL21(EadM) were dramatically decreased (approximately 10,000-fold) at 4, 8, and 24 h after 1 mM IPTG treatment compared to BL21(EV). We also confirmed the presence of EadM by immunoblotting after induction in BL21(EadM), which was not observed in BL21(EV) (Fig. 6C).

## Discussion

It is generally known that DNA methyltransferases influence the cell growth rate by affecting replication mechanisms in bacteria. For example, a knockout mutant of M.NgoAX, a DNA methyltransferase, showed enhanced bacterial growth velocity in *Neisseria gonorrhoeae*^27^. However, growth patterns of *Xag*(EadM) in both minimal and rich media were similar to those of *Xag*(EV) (Fig. 1C), indicating that EadM does not significantly affect DNA replication or cell division. Overexpression of EadM was negatively involved in virulence on soybean (Fig. 1A). Thus, reduced virulence of *Xag*(EadM) is related to other mechanisms, but not cell division. In *Helicobacter pylori*, DNA methyltransferase, M2.HpyAll is related to transcription as well as virulence^28^. Similar to overexpression of EadM in *Xag*, in *Photorhabdus luminescens*, a lethal pathogenic bacterium of insects, the strain overexpressing Dam DNA methyltransferase showed significantly decreased motility and virulence^29^. Interestingly, *Xag*(EadM) produced unstable cell envelopes compared to *Xag*(EV) (Fig. 3). The unstable, abnormal cell envelopes may interfere with light scattering during OD value measurement, showing reduced viable cell numbers at same OD values compared to *Xag*(EV) (Fig. 1B). In a previous study, cell envelope stress responses were found to be crucial for regulating bacterial virulence gene expression^30^. Therefore, functions of EadM may be associated with bacterial cell envelopes integrity, which may contribute the virulence of *Xag* on soybean. Comparative proteomic analysis supported that EadM is involved in cell envelope functions because the most abundant proteins affected by EadM were in group M (cell wall/membrane/envelope biogenesis).

In gram-negative bacteria, the bacterial cell envelope is the outermost multilayered structure that protects cells from the external environment^22^. Because of alterations to the cell envelope of *Xag*(EadM), the strain showed reduced viability following exposure to external factors including ciprofloxacin and sorbitol (Fig. 5A,B). Following exposure to ciprofloxacin and sorbitol, unstable envelopes in *Xag*(EadM) may have increased the sensitivity to both conditions compared to in *Xag*(EV). In an agreement with our observations, *Vibrio cholerae* lacking VchM protein (DNA methyltransferase) exhibited unstable cell envelopes and decreased bacterial growth in LB containing antibiotics polymyxin B^31^. Taken together, diverse DNA methyltransferases are crucial for bacterial cell envelope functions and tolerance to antibiotic agents. It is well-known that plants produce antimicrobial compounds or phytotoxic materials to protect themselves against pathogen infection and pathogens must overcome these conditions for successful infection^32^. Therefore, reduced tolerance to external factors in *Xag*(EadM) may contribute to decreased virulence.

Proteome analysis showed that the abundance of cell wall/membrane/envelope-related proteins was affected by EadM. These proteins are known to be involved in outer membrane structures including EPS biosynthesis^33,34^. In a nosocomial pathogen, *Klebsiella pneumoniae* causing urinary tract infections and pneumonia, the bacterial capsule polysaccharide was crucial for resistance to antimicrobial peptides^35^. Similarly, *Xag*(EadM) also displayed reduced EPS production compared to *Xag*(EV) (Fig. 5C), suggesting that EPS production is influenced by unstable envelopes in *Xag*(EadM). Under desiccation conditions, *Xag*(EadM) showed higher viability than *Xag*(EV) (Fig. 4D). In a previous study, mucoid strains of bacteria showed significant resistance to desiccation compared to nonmucoid strains^25^. Similarly, peeled and accumulated envelopes in *Xag*(EadM) may protect the bacterium from desiccation by protecting living cells under the given condition. In addition, the abundance of proteins related to iron-related mechanisms was affected by EadM expression in *Xag* (Supplementary Tables 2 and 3). In hosts, pathogenic bacteria encounter iron-restricted condition because of the presence of unusable forms of iron^36^ and strive to maintain iron homeostasis because iron is essential for bacterial growth and viability^37,38^. *Xanthomonas* spp. produce siderophores for iron uptake^39^. Neither *Xag*(EV) nor *Xag*(EadM) produced siderophores in the presence of iron, while *Xag*(EadM) displayed higher siderophore production compared to *Xag*(EV) under iron-deficient conditions (Fig. 5). Because the secretion of siderophores and iron uptake are closely related to the stability of the bacterial cell wall/membrane/envelope^40^, abnormal and unstable envelopes in *Xag*(EadM) may have affected siderophore production.

*Xag* and *E. coli* are gamma-proteobacteria. Unexpectedly, the effects of EadM expression in *E. coli* were more severe than those in *Xag*. After expression of EadM, the viable cell numbers in *E. coli* were significantly reduced (approximately 1000-fold) compared to those in other controls (Fig. 6), but the viable cell numbers in *Xag*(EadM) were decreased by only 10-fold, indicating that the mechanisms associated with EadM are conserved in both species and that *E. coli* BL21 is more sensitive than *Xag* to expression of EadM. Alternatively, EadM protein induced by IPTG in BL21 was likely more abundant than this in *Xag*(EadM), resulting in severely reduced viable cell numbers in BL21. The protein displays high homology (over 81% identity) with putative site-specific DNA methyltransferases in other bacteria belonging to the order Xanthomonadales (Supplementary Fig. 1), but only 23% identity with a homolog (Accession. No. NP_417728) in *E. coli* BL21 (data not shown). This suggests that the functions of EadM evolved to be specific to Xanthomonadales. Because EadM is a putative site-specific DNA methyltransferase and overexpression of EadM causes similar effects in both *Xag* and *E. coli*, the motif is likely conserved in both species. Therefore, we attempted to identify the putative methylation motif by the single molecule real time (SMRT) sequencing and *E. coli* ER3413, which is a DNA methyltransferase-deficient strain and was used to identify the DNA methylation motif^41^. Methylomes from ER3413 carrying an empty vector and ER3413 expressing EadM were analyzed and compared using a previously established protocol^8^. However, this was not successful and the motif recognized by EadM was not assigned. We postulate reasons for the failure that the current analysis technique is limited, that the strain still carries a DNA methyltransferase whose motif is identical with EadM, or that the strain does not have a putative motif for EadM. If the motif is identified in the near future, it can be determined how EadM controls cellular and biological mechanisms.

Proteomic analysis can be applied in diverse types of research. For example, protein expression profiling reveals mechanisms related to disease and structural proteomics can also provide the information regarding protein complexes^42^. In addition, putative proteins related to virulent mechanisms were identified in three *Xanthomonas* spp. using a label-free shotgun proteomic technique^43^. Comparative proteomics used in this study corroborated the functions of the protein by comparing protein expression patterns in *Xag*(EV) and *Xag*(EadM). We performed phenotypic assays based on the proteomic analysis. The results of diverse phenotypic assays agreed with our predictions. Using a similar approach, we also determined the functions of diverse proteins related to virulence in *Xanthomonas* spp.^44,45^. Moreover, two lineages of *Mycobacterium tuberculosis* were evaluated by comparative proteomic analysis which revealed that differentially abundant proteins are linked to growth and virulence^46^. Thus, a combination of proteomic analysis and phenotypic characterization enabled determination and functional characterization of genes/proteins in biological processes.

In this study, the roles of EadM, a putative DNA methyltransferase, in *Xag* and its related biological and cellular mechanisms were predicted by label-free shotgun comparative analysis and COG categorization. Subsequently, we validated and tested its functions through diverse phenotypic assays. Using these approaches, we confirmed that EadM affects the stability of bacterial cell wall/envelopes, tolerance to various stresses, and production of EPS and siderophore, which may contribute to the virulence of *Xag*. Finally, we demonstrated that EadM-related mechanisms may be conserved in *Xag* and *E. coli*. Our results provide insights into the functions of a DNA methyltransferase in plant pathogenic bacteria.

## Methods

### Bacterial strains and growth conditions

*Xanthomonas axonopodis* pv. *glycines* (*Xag*) strain 8ra^8^ was grown in TSB (Tryptic Soy Broth Soybean-Casein Digested, 30 g/L) or XVM2 (20 mM NaCl, 10 mM (NH_4_)_2_SO_4_, 5 mM MgSO_4_, 1 mM CaCl_2_, 0.16 mM KH_2_PO_4_, 0.32 mM K_2_HPO_4_, 0.01 mM FeSO_4_, 10 mM fructose, 10 mM sucrose, and 0.03% casamino acids (pH 6.7)^47^ at 28 °C. *Escherichia coli* DH5α for the proliferation of plasmids and BL21 for protein expression were grown in LB (Luria Bertani; 1% tryptone, 0.5% yeast extract and 1% NaCl) at 37 °C. For selection, the antibiotics cephalexin (30 μg/mL), gentamicin (10 μg/mL), and ampicillin (100 μg/mL) were used in this study.

### Generation of *Xag* strain 8ra overexpressing EadM

To produce the construct for generating the EadM-overexpressing mutant, the open reading frame was amplified using EadM-specific primers, 5′-ctcgagatgaaaaaccagctctgca-3′ and 5′-gtagccgaatctgcgaaattcaccaccaccaccaccactgaagctt-3′. The amplified fragment was cloned into the pGem T-easy vector (Promega, Madison, WI, USA) and the sequence was confirmed by Sanger sequencing. The confirmed fragment was digested with *Xho*I and *Hind*III and the excised fragment was cloned again into pBBR1-MCS5, which is a broad host range vector and contains *lac* promoter for expression^48^, creating pBRR1-EadM. The pBRR1-EadM was introduced into the wild-type by Bio-Rad Micropulser^TM^ (Bio-Rad, Hercules, CA, USA) and the transformant was selected on TSA plates containing the gentamycin and confirmed by PCR. The selected overexpression strain was designated as *Xag*(EadM). In addition, the empty vector was introduced into *Xag*, producing *Xag*(EV) as a negative control.

### Quantitative PCR

*Xag* strains were incubated in TSB and harvested at an optical density of 0.6 at 600 nm (OD_600 nm_). After extraction of RNA using a High Pure RNA Isolation Kit (Roche, Mannheim, Germany), cDNA was synthesized by a RevertAid First Strand cDNA Synthesis Kit (Thermo Fisher Scientific, Rockford, IL, USA). Four EadM primer sets (1: 5′-ccaagtactgccgagatggt-3′ and 5′-acacgtgcgcactcagatag-3′, 2: 5′-aaggctgacaagcatcacct-3′ and 5′-tccagcgataaccctcaagt-3′, 3: 5′-cacgtgtgcttaaagacggc-3′ and 5′-tcggtcttatcccagacggt-3′, 4: 5′-cgaccaagtactgccgagat-3′ and 5′-acacgtgcgcactcagatag-3′) were used to check gene expression, and 16 S RNA primers were employed as the reference gene for normalization. The qPCR was performed with an IQ™ SYBR Green Supermix (Bio-Rad, Hercules, CA, USA) on a CFX connect™ (Bio-Rad, Hercules, CA, USA). The experiment with three replicates was repeated at least twice. The *ΔΔ*Ct method was used for the calculation of gene expression levels.

### Virulence assay

*Glycine max* cv. Jinju1 plants were grown in controlled chambers for 3 weeks. To prepare inoculums, *Xag* strains were grown in TSA for 48 h, suspended in 10 mM MgCl_2_ strains to 0.3 at OD_600 nm_, and diluted (10^−3^) with 10 mM MgCl_2_ which corresponds to 10^5^ colony forming unit (CFU)/mL. In the case of 10 × *Xag*(EadM), the inoculum was less diluted (10^−2^). The diluted inoculums were infiltrated into fully expanded trifoliate leaves using needleless syringes^49^. To monitor bacterial growth, the infiltrated leaves were punched with cork-borers (0.4 cm in a diameter) and two leaf discs were ground in 200 μL of sterilized water. The extracted bacterial cells were serially diluted and dotted onto TSA containing appropriate antibiotics. Three biological replicates were used for the assay.

### Measurement of viable cell numbers and establishment of growth curve

To evaluate viable cell numbers following expression of EadM, *Xag* and *E. coli* strains were grown on TSA and LB plates, respectively. After harvesting the bacterial cells, the cells were washed twice, resuspended in sterilized water, and adjusted to various concentrations (0.005, 0.01, 0.05, 0.1, 0.5, or 1) at OD_600 nm_ using a spectrophotometer, OPTIZEN POP (MECASYS, Daejeon, Korea). After serial dilution, the number of viable cells was determined using a colony counting method. To verify the effects of EadM on bacterial growth, we monitored the growth patterns of *Xag* strains using TSB and XVM2. The bacterial suspension was adjusted to 0.3 at OD_600 nm_ and serially diluted (10^−3^) with media, after which OD_600 nm_ values were monitored for 5 days at 12-h intervals. Three biological replicates were used for this assay.

### Label-free shotgun proteomic analysis

Detailed processes and conditions, including the extraction of total proteins, preparation of peptides, liquid chromatography-tandem mass spectrometry, identification and quantification of peptides, comparison of protein abundance with statistical analysis, and clusters of orthologous group (COG) categorization, were conducted as described previously^43^. Briefly, *Xag* strains with three biological replicates were grown in XVM2 and harvested by centrifugation when the OD_600 nm_ value reached 0.6. After protein extraction and peptide generation, the samples were analyzed with a split-free nano LC system (EASY-nLC II; Thermo Fisher Scientific, Waltham, MA, USA) connected to an LTQ Velos Pro instrument (Thermo Fisher Scientific). Obtained mass spectra were identified using the *Xag* strain 8ra database from the National Center for Biotechnology Information and the peptides were quantified with Thermo Proteome Discoverer 1.3 (ver. 1.3.0.399) combined with the SEQUEST program. After identification and quantification of the peptides, we compared protein abundance in *Xag*(EV) and *Xag*(EadM). Finally, differentially abundant proteins were classified by COG analysis.

### Scanning electron microscopy

The bacterial strains were incubated for 24 h at 28 °C on a rotary shaker (200 rpm). The culture was filtered by though a 0.2-μm polycarbonate membrane (Whatman International, Ltd., Maidstone, UK). The bacterial cells were post-fixed in 1% osmium tetroxide solution (Sigma-Aldrich, St. Louis, MO, USA) in 0.1 M phosphate buffer (pH 7) at room temperature for 2 h^50^. The samples were washed 3 times in 0.1 M phosphate buffer and then dehydrated in gradient of ethanol (30, 50, 70, 80, 90, and 100%, once for concentrations up to 90% and 3 times for the 100% concentration) by incubation for 10 min in each concentration. The samples were placed in a critical point dryer (VTRC-620, Jeio Tech Co., Daejeon, Korea) to complete dehydration and sputter-coated with platinum in a Cressington 108 auto Sputter Coater (Cressigton Scientific Instruments, Ltd., Watford, UK) for 90 s^51^. Samples were observed by scanning electron microscopy with a JSM-6700F microscope (Jeol, Tokyo, Japan).

### Tolerance assay

To estimate the roles of EadM in stress tolerance, we used various stress factors including ciprofloxacin, D-sorbitol, and desiccation^52^. The bacterial suspensions were adjusted to 0.1 at OD_600nm_ and the survival of *Xag* cells was examined against three stress factors. *Xag* strains were exposed to 0, 0.1, 0.5, 1, 2, and 10 μg/mL of ciprofloxacin for 2 h and 40% D-sorbitol for 20 m in TSB. After treatment, the cultures were serially diluted with sterilized water and bacterial numbers were determined by a spread plate counting method. The half maximal inhibitory concentration (IC50) was calculated by the Prism8 program (GraphPad, San Diego, CA, USA) For desiccation, 100 μL of *Xag* suspensions were dropped onto a cover glass under aeration conditions on a clean bench and incubated for 1, 2, and 3 h at room temperature. *Xag* cells were recovered from dried samples using 1 mL of 10 mM MgCl_2_ and bacterial numbers were established by a colony counting method. Cell viability was calculated as the ratio of bacterial numbers before treatment to those after treatment.

### EPS analysis

To determine whether EadM is involved in EPS production, we used a previously reported protocol with some modifications^24^. After harvesting the *Xag* strains, the cells were diluted in TSB to 0.1 at OD_600 nm_ and incubated at 28 °C for 5 days. After collecting the supernatants by centrifugation, 400 μL of the supernatant was mixed with 1.2 mL of EtOH and the mixture was placed at −20 °C. On the following day, the pellet was collected by centrifugation (16,500 × *g*) for 10 min at 4 °C and dried on the clean bench. Dried samples were resuspended in 1 mL of sterilized water and 100 μL of samples were diluted with 900 μL of sterilized water. Next, 5 mL of sulfuric acid and 1 mL of aqua phenol (5%) were added to the diluted samples and OD values were measured at 488 nm using a spectrophotometer. Three biological replicates were used for the assay.

### Chrome azurol sulfonate assay

To investigate siderophore production, the chrome azurol sulfonate (CAS) assay was used^53^. *Xag* strains were cultured on XVM2-CAS-agar, an iron-rich condition, and XVM2-CAS-bipyridyl (BP)-agar plate, an iron-deficient condition, for 3 days. BP was used at a final concentration of 100 μM. The cultured cells were washed three times with sterilized water. *Xag*(EV) and *Xag*(EadM) were diluted with sterilized water to 0.3 at OD_600 nm_ and 10 × *Xag*(EadM) was less (10-fold) diluted. Three microliters of bacterial cells were dropped onto XVM2-CAS-agar and XVM2-CAS-BP-agar plates and colony and halo diameters were measured. Three biological replicates were used for the assay.

### Expression of EadM in *E. coli* BL21

To express EadM in the *E*. *coli* strain BL21, the open reading frame of *eadM* was amplified using pOPINF-EadM-specific primers with primers 5′-aagttctgttcagggcccgaaaaaccagctcctgcaggg-3′ and 5′-atggtctagaaagctttattaaatttcgcagattcggc-3′. The amplified fragment was cloned into the pOPINF vector using the In-Fusion cloning kit (Clontech, Mountain View, CA, USA), creating pOPINF-EadM. The construct was introduced into *E*. *coli* BL21 by electroporation, generating the BL21(EadM) strain. BL21(EV) was also created using the pOPINF as a negative control. One millimolar IPTG was used to induce expression of EadM in BL21 cells. To measure viable cell numbers of BL21 strains at various OD values, identical methods were used for *Xag* strains. For time course expression, the BL21(EadM) and BL21(EV) strains were collected at 0, 4, 8, and 24 h after adding 1 mM IPTG and the viable cell numbers were evaluated by a colony counting method. The expression of EadM was confirmed by western blotting using an anti-6xHis antibody.

### Statistical analysis

All experiments were repeated at least three times with three biological replicates. Statistical analyses were conducted by performing a *t*-test and one-way analysis of variance combined with Tukey’s multiple comparison using SPSS 12.0 K software (SPSS, Inc., Chicago, IL, USA). A P-value less than 0.05 was considered to indicate a significant difference.

## Supplementary information

Supplementary Figure 1-4 and table 1

Supplementary Table 2

Supplementary Table 3
