# Supplementary material for: Functional characterization of a putative DNA methyltransferase, EadM, in Xanthomonas axonopodis pv. glycines by proteomic and phenotypic analyses
Source: Sci Rep. 2019 Feb 21;9:2446. doi: 10.1038/s41598-019-38650-3 (PMC6385262; doi:10.1038/s41598-019-38650-3)
Supplement: Supplementary file 1 — Supplementary Figure 1-4 and table 1 [file 41598_2019_38650_MOESM1_ESM.pdf]

Supplementary information for

Functional characterization of a putative DNA methyltransferase, EadM, in *Xanthomonas axonopodis* pv. *glycines* by proteomic and phenotypic analyses

Hye-Jee Park[1], Boknam Jung[2], Jungkwan Lee[2], and Sang-Wook Han[1,*]

[1]Department of Integrative Plant Science, Chung-Ang University, Anseong, 17546, Republic of Korea. [2]Department of Applied Biology, Dong-A University, Busan 49315, Republic of Korea

*Corresponding author

Sang-Wook Han

E-mail: swhan@cau.ac.kr

Supplementary information includes

Supplementary Figure 1-4

Supplementary Table 1

*Supplementary Table 2 and 3 were provided in the separated files

```
EZP46960        MKNQLIHGDALTVLPTLPAASFDALITDPPYASGGVHAASRQQAPSSKYVRSG---LHDD  57
EadM            MKNQLLQGDALTILPTLEADSFDALITDPPYASGGLTAAARARPPSTKYCRDGG---HAD  57
WP_031421399    MKNQLLQGDALTILPTLEANSFDALITDPPYASGGLTAAARARSPSTKYCRDGG---HAD  57
SOD53705        MKNQLLKGDALTLLPTLEACSFDALITDPPYASGGLTAGARQKPPSEKYVQGGKSALHAD  60
                *****::*****:**** * ***************: *.:* : ** ** :.*    * *

EZP46960        FVGDERDQRSHLAWMRLWLAQCSRVLKDGAPVLLFTDWRQLPLTTDALQCAGFTWRGVAV  117
EadM            FVGDERDQRSHLKWMHLWLSECARVLKDGAPVLLFTDWRQLPLTTDALQIAGFTWRGITV  117
WP_031421399    FVGDERDQRSHLKWMHLWLSECARVLKDGAPVLLFTDWRQLPLTTDALQIAGFTWRGITV  117
SOD53705        FAGDERDQRSHLRWMVMWLSECARLLKEGAPVCLFTDWRQLPLTTDALQCAGFTWRGITV  120
                *.********** ** :**::*:*:**:**** **************** *******::*

EZP46960        WDKTEGVRPQLGRFRNQAEYVVWGSKGNMPLGRRAPVLPGVIRESVRKADKHHMTGKPTD  177
EadM            WDKTEGVRPQLGRFRNQAEYIVWGSKGNMPLDRRAPVLPGVIREPVRKADKHHLTGKPTE  177
WP_031421399    WDKTEGVRPQLGRFRNQAEYIVWGSKGNMPLDRRAPVLPGVIRESVRKADKHHLTGKPTE  177
SOD53705        WDKTEGVRPQLGRFRNQAEYVVWGSKGSMPLQRRAPVLPGVIREPVRKADKLHMTGKPTA  180
                ********************:******.*** ************ ****** *:*****

EZP46960        LMRQLVRICEEGGRILDPFAGSGTTLVAADQEGYSWTGIEMTEHYFGVAERRLPSE      233
EadM            LMRRLVRICESGGCVLDPFAGSGTTLVAAELEGYRWTGVEKTEHYATVAGSRICEI      233
WP_031421399    LMRQLVRICEAGGRVLDPFAGSGTTLIAAQLEGYNWTGVEITRHYASSAISRLSEL      233
SOD53705        LMRQLVRICEEGGRVLDPFAGSGTTLVAAQLEGYSWLGCEMTDHYVEVAGQRLAAL      236
                ***:****** ** :***********:**: *** * * * * **   *  *:
```

Supplementary Figure 1. Sequence alignment of EadM and its homologs. The deduced amino acids of EadM were compared with its homologs in other bacteria belonging to the order Xanthomonadales, using the CLUSTALW program. EZP4690, WP_031421399, and SOD53705 are putative site-specific DNA methyltransferases in *Stenotrophomonas* sp. RIT309, *Xanthomonas euvesicatoria*, and *Pseudoxanthomonas wuyuanensis*, respectively. ‘*’, ‘:’, and ‘.’ indicate identical residues, conserved substitutions, and semi-conserved substitutions, respectively.

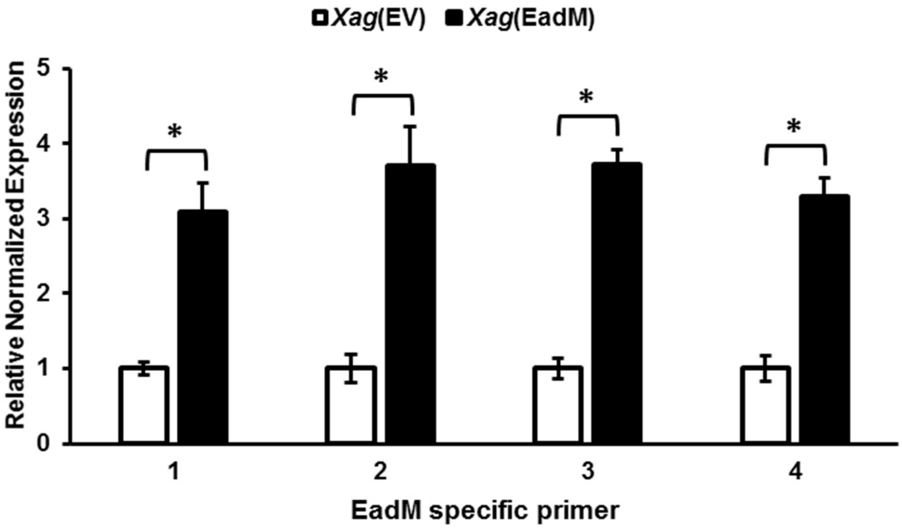

Supplementary Figure 2. Relative expression of *eadM* gene in *Xag* and *Xag*(EV). *Xag* strains were harvested at 0.6 in an $OD_{600\ nm}$ and total RNAs were extracted. The four *eadM*-specific primer sets were used for quantitative PCR. The 16S rRNA gene was used as a reference. Bars are the mean of three replicates ± standard deviation. The asterisk indicates the statistical difference (t-test, $P < 0.05$).

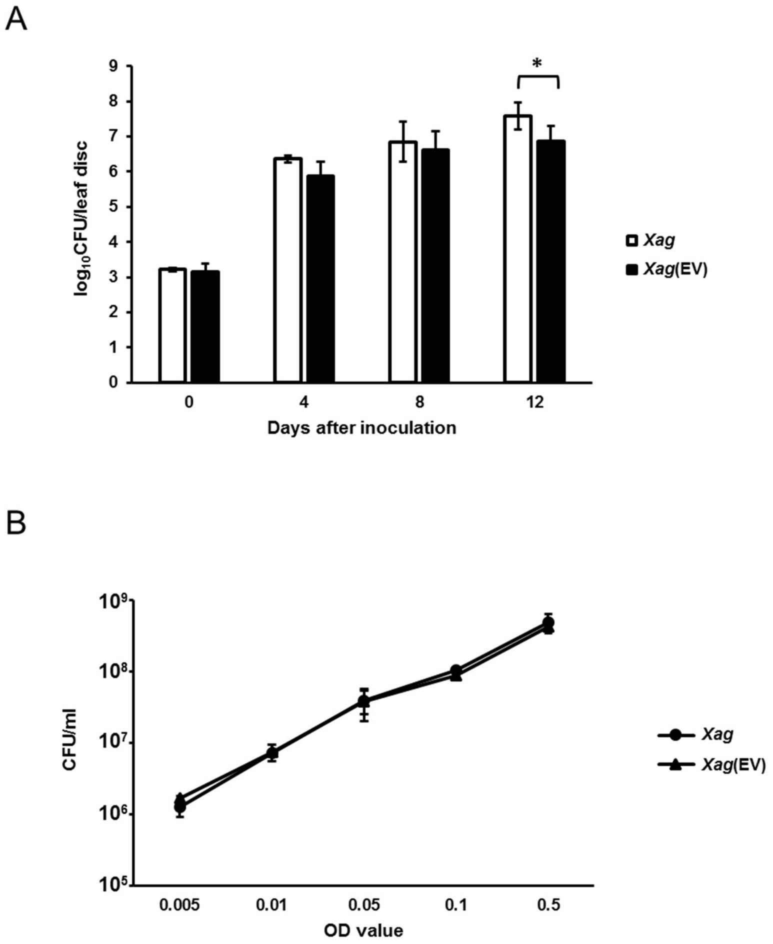

Supplementary Figure 3. Measurement of bacterial population in plants and viable cell numbers at different optical density values for *Xag* and *Xag*(EV). (A) Bacterial population of *Xag* (white), and *Xag*(EV) (black) were determined by the colony counting method at 0, 4, 8, and 12 days after inoculation. (B) The viable cell numbers of *Xag* (circle) and *Xag*(EV) (triangle) were quantified by the colony counting method at various OD values using a spectrophotometer. Different letters represent significant differences using the least significant difference test, $P \leq 0.05$. Error bars represent the mean of three biological replicates with the standard deviations. All experiments were repeated at least three times with three biological replicates.

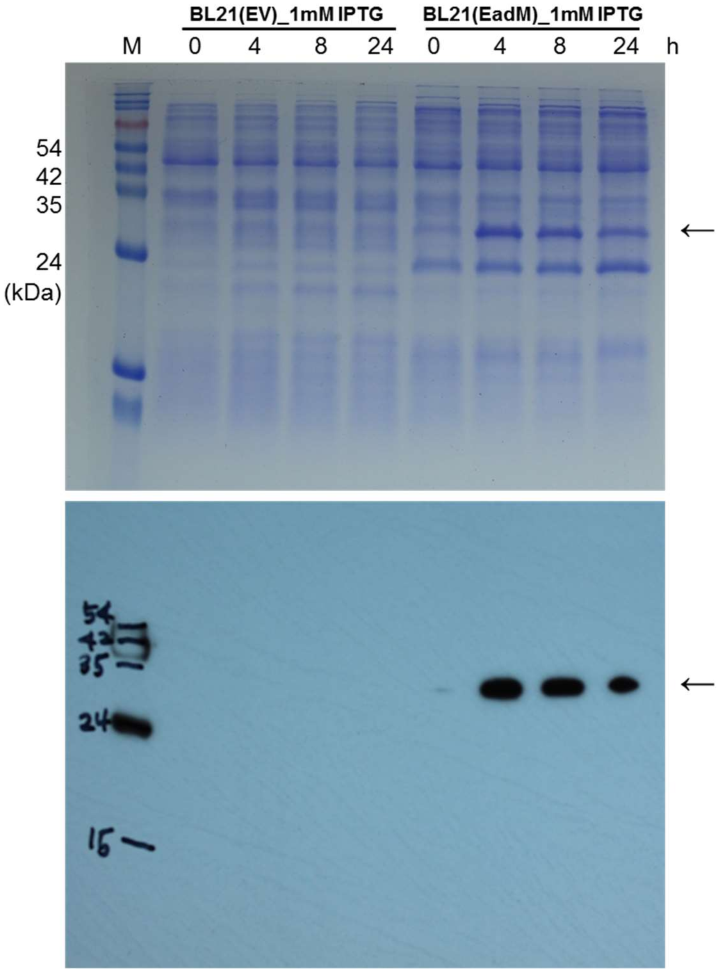

Supplementary Figure 4. The full-length gel and blot used in Figure 6C. Arrows indicate EadM protein.

Supplementary Table 1. Proteins and peptide spectral matches between *Xag*(EV) and *Xag*(EadM) in three biological replicates from LC-MS/MS

Strains	1st		2nd		3rd		shared proteins in 3 biological replicates
	protein	PSM	protein	PSM	protein	PSM	
Xag(EV)	1100	47408	1077	47425	1087	47415	1029
Xag(EadM)	1140	50892	1148	50910	1154	50923	1071
